# Supplementary material for: An interbacterial DNA deaminase toxin directly mutagenizes surviving target populations
Source: eLife. 2021 Jan 15;10:e62967. doi: 10.7554/eLife.62967 (PMC7901873; doi:10.7554/eLife.62967)
Supplement: Supplementary file 2. [file elife-62967-supp2.docx]

|  | ***P. syringae* SsdA_tox_ / SsdA_I_ SAD peak^a^** |
| --- | --- |
| PDB accession code | 7JTU |
| **Data Collection** |  |
| Space group | I4_1_22 |
| Cell dimension  *a, b, c* (Å) | 93.07, 93.07, 383.49 |
| a, b, g (°) | 90, 90, 90 |
| Wavelength (Å) | 0.9790 |
| Resolution (Å) | 33.4 - 3.0 (3.15-3.0)^b^ |
| No. unique reflections | 17597 (434) |
| Rmerge | 0.12 |
| I/sI | 22.9 (2.7) |
| Completeness (%) Total | 100 (100) |
| Anomalous | 100 (100) |
| Redundancy | 28.6 (29.6) |
| Wilson B-factor (Å^2^) | 79.6 |
| **Refinement** |  |
| Resolution (Å) | 33.4 – 3.0 (3.1 – 3.0) |
| No. reflections | 17248 (1193) |
| Rwork/Rfree (%) | 20.2 / 23.5 (32.3 / 40.5) |
| No. atoms  Protein | 2717 |
| Ligand/ion | 0 |
| Water | 3 |
| B-factors (Å^2^) Protein | 79.6 |
| Ligand/ion | N/A |
| Water | 63.9 |
| rmsd  Bond lengths (Å) | 0.009 |
| Bond angles (°) | 1.086 |
| Missing residues chain A | 245-257, 409 |
| chain B | none |

^a^All data collected from a single crystal

^b^Values in parentheses are for the highest resolution shell
